# Up-conversion luminescence, temperature sensing properties and laser-induced heating effect of Er^3+^/Yb^3+^ co-doped YNbO_4_ phosphors under 1550 nm excitation

**DOI:** 10.1038/s41598-018-23981-4

**Published:** 2018-04-10

**Authors:** Xin Wang, Xiangping Li, Hua Zhong, Sai Xu, Lihong Cheng, Jiashi Sun, Jinsu Zhang, Lei Li, Baojiu Chen

**Affiliations:** College of Science, Dalian Maritime University, Dalian, Liaoning 116026 PR China

## Abstract

YNbO_4_ phosphors with various Er^3+^ and Yb^3+^ concentrations were synthesized via a traditional high-temperature solid-state reaction method. Their crystal structure was investigated by means of X-ray diffraction (XRD) and Rietveld refinements, and it was confirmed that the obtained samples exist in monoclinic phase. The Er^3+^ and Yb^3+^ concentration-dependent up-conversion (UC) luminescence was studied under 1550 nm excitation. By inspecting the dependence of UC intensity on the laser working current, it was found that four-photon and three-photon population processes were co-existent for generating the green UC emissions in the samples with higher Yb^3+^ concentrations. In addition, it was observed that the temperature sensing properties of YNbO_4_: Er^3+^/Yb^3+^ phosphors were sensitive to both Er^3+^ and Yb^3+^ doping concentrations. Furthermore, based on the obtained temperature response of the UC luminescence phosphors, 1550 nm laser-irradiation-induced thermal effect was studied, and it was discovered that the sample temperature was very sensitive to the doping concentrations of Er^3+^ and Yb^3+^ and the excitation power.

## Introduction

Rare-earth (RE) ion-doped up-conversion (UC) luminescence materials have received considerable attention due to their widespread applications in many fields, such as UC lasers, sensors, solar cells, three dimension display and so on^1,2^. In practical applications, the UC phosphors with high luminescence efficiency and reliable temperature sensing ability may be preferable. Nevertheless, the UC materials possessing both highly efficient UC efficiency and high temperature sensitivity are very fewer^3^. Therefore, it is necessary to explore a new type material to meet this requirement.

On the one hand, choosing proper trivalent RE ions used as absorption and emission centers in the design of UC luminescence materials is very important in order to obtain highly efficient UC emissions^4–6^. Among trivalent RE ions, Er^3+^ is one of the most preferable RE ions for UC luminescence due to its uniform energy level distribution and proper energy distance between some metastable energy levels, which allows Er^3+^ to be effectively excited by different laser wavelengths, such as 800, 980 and 1550 nm^7^. Moreover, Er^3+^ is expected as an effective temperature sensing unit with high temperature sensing sensitivity based on its two thermally coupled emitting energy levels ^2^H_11/2_ and ^4^S_3/2_, whose energy distance is about 770 cm^−1 ^^8,9^. In order to further enhance the UC luminescence efficiency of Er^3+^, Yb^3+^ is often employed as a sensitizer owing to its large absorption cross-section at around 980 nm and efficient energy transfer to Er^3+ ^^10^. Thus, Er^3+^/Yb^3+^ co-doped UC luminescence phosphors have been extensively studied^4–6,8,9^. On the other hand, the characteristics of the host materials, such as their phonon energy, chemical and thermal stabilities, have significant effects on the UC luminescence properties of the UC phosphors^11–14^. In general, oxide compounds have good chemical and physical stabilities, which are beneficial to the applications in high temperature environments, have received increasing attention in recent years^15–22^. Among various oxide matrixes, ANbO_4_ (A = Gd, Y, La) compounds, with a fergusonite structure and low phonon energy, are good host candidates for UC luminescence phosphors^17–22^. Recently, the UC luminescence properties of Er^3+^/Yb^3+^ co-doped ANbO_4_ materials under 980 nm excitation have been reported^17,18^. It was found that the ANbO_4_ host presented high chemical and physical stabilities, low lattice vibration energy and high accommodating concentration for RE luminescent centers; additionally they can be easily prepared via the traditional solid-state reaction method. However, the UC luminescence properties reported in these niobate systems are mainly focused on the 980 nm excitation. In addition to 980 nm excitation, the Er^3+^ can also be excited by 1550 nm. 1550 nm laser has obtained considerable attention due to its eye-safe nature and wide applications in optical communication and solar cells; meanwhile, it is also an effective excitation source to investigate the UC luminescence properties of RE^3+^-doped materials^23–25^. It is reported that efficient UC emission from Er^3+^ was obtained under monochromatic excitation around 1500 nm^24^. Previously, we have studied the UC luminescence properties of Er^3+^ in Er^3+^ mono-doped YNbO_4_ phosphors under 1550 nm excitation^22^. The results showed that the relative intensity of the green and the red UC emissions were significantly different in down-conversion from that in UC luminescence processes because of the different population routes and pumping conditions. Furthermore, it was confirmed that energy transfer process was the main mechanism for UC emissions of Er^3+^ in Er^3+^ mono-doped YNbO_4_ phosphors excited at 1550 nm. In addition, we have also studied the effect of Mo^6+^ on the UC luminescence and temperature sensing sensitivity of Er^3+^ in ANbO_4_ phosphors excited at 1550 nm^20^. However, there is little work on the study of UC luminescence, temperature sensing property and laser-induced thermal effect of Er^3+^/Yb^3+^ co-doped ANbO_4_ phosphors under 1550 nm excitation.

Hereby, in this study, Er^3+^/Yb^3+^ co-doped YNbO_4_ phosphors were prepared by a traditional high-temperature solid-state reaction method. Er^3+^ and Yb^3+^ concentration-dependent UC luminescence processes under 1550 nm excitation were discussed. Different concentration dependent UC luminescence processes for the green and red UC emissions were discovered. In addition, the effects of Er^3+^ and Yb^3+^ concentrations on the optical temperature sensing properties were also investigated according to fluorescence intensity ratio (*FIR*) technique. Furthermore, the laser-irradiation-induced thermal effect was evaluated by persistent irradiation of the 1550 nm laser. It was observed that both the temperature sensing property and laser-induced thermal effect were dependent on the Er^3+^ and Yb^3+^ concentrations.

## Experimental Details

### Sample preparation

Usually, doping concentration has significant effect on the luminescent performance of the UC luminescence materials. Previously, we have investigated the spectroscopic properties of Er^3+^ mono-doped YNbO_4_ phosphor and obtained the optimum Er^3+^ concentration (5 mol%)^22^. According to our previous results^16,25^, two sets of YNbO_4_: Er^3+^/Yb^3+^ phosphors were prepared by a traditional high-temperature solid-state reaction method. In one set, the concentration of Er^3+^ was fixed to be 5 mol%, and the doping concentrations of Yb^3+^ were designed to be 0, 2, 6, 10, 14, 16 and 20 mol%. In the other set, the concentrations of Er^3+^ were respectively set as 0.5, 1, 3, 5, 7, 9, 12 and 15 mol%, and the concentration of Yb^3+^ was fixed to be 6 mol%, which was the optimum doping concentration derived from the experimental results of the first set of the samples.

The starting materials Y_2_O_3_ (99.99%), Yb_2_O_3_ (99.99%) and Er_2_O_3_ (99.99%) used in this work were purchased from Shanghai Second Chemical Reagent Factory (China), Nb_2_O_5_ (99.99%) were purchased from Tianjin Reagent Chemicals Co., Ltd (China). They were first weighed according to the designed stoichiometric ratio and fully ground in an agate mortar for 30 min until the mixtures were uniformly mixed. Furthermore, the mixture was transferred into a crucible, and then it was put into a muffle furnace (SX-GO 3163, Tianjin Zhonghuan Experimental Electric Furnace Co., Ltd., China) and calcined at 1300 °C for 4 h. Last, the final products were obtained after the furnace was naturally cooled to room temperature.

### Sample characterization

A SHIMADZU X-ray diffractometer (XRD-6000) with CuKα_1_ radiation (λ = 0.15406 nm) was used to analyze the crystal structure of those as-prepared samples. Temperature dependent UC luminescence spectra in the visible light region were measured by a Hitachi F-4600 fluorescent spectrometer equipped with a power-controllable 1550 nm fiber laser as an excitation source. The output power of the 1550 nm fiber laser shows a linear dependence on its working current. The fluorescence spectra in the wavelength range of 800–1100 nm were obtained by a near-infrared spectrometer (NIR QUEST-256) with an external 1550 nm fiber laser. The temperatures of the samples were controlled by a self-made sample temperature controlling system, DMU-TC 450, with a controlling accuracy of about ±0.5 °C.

## Results and Discussion

### Identification of the crystal structure

**Figure 1:**
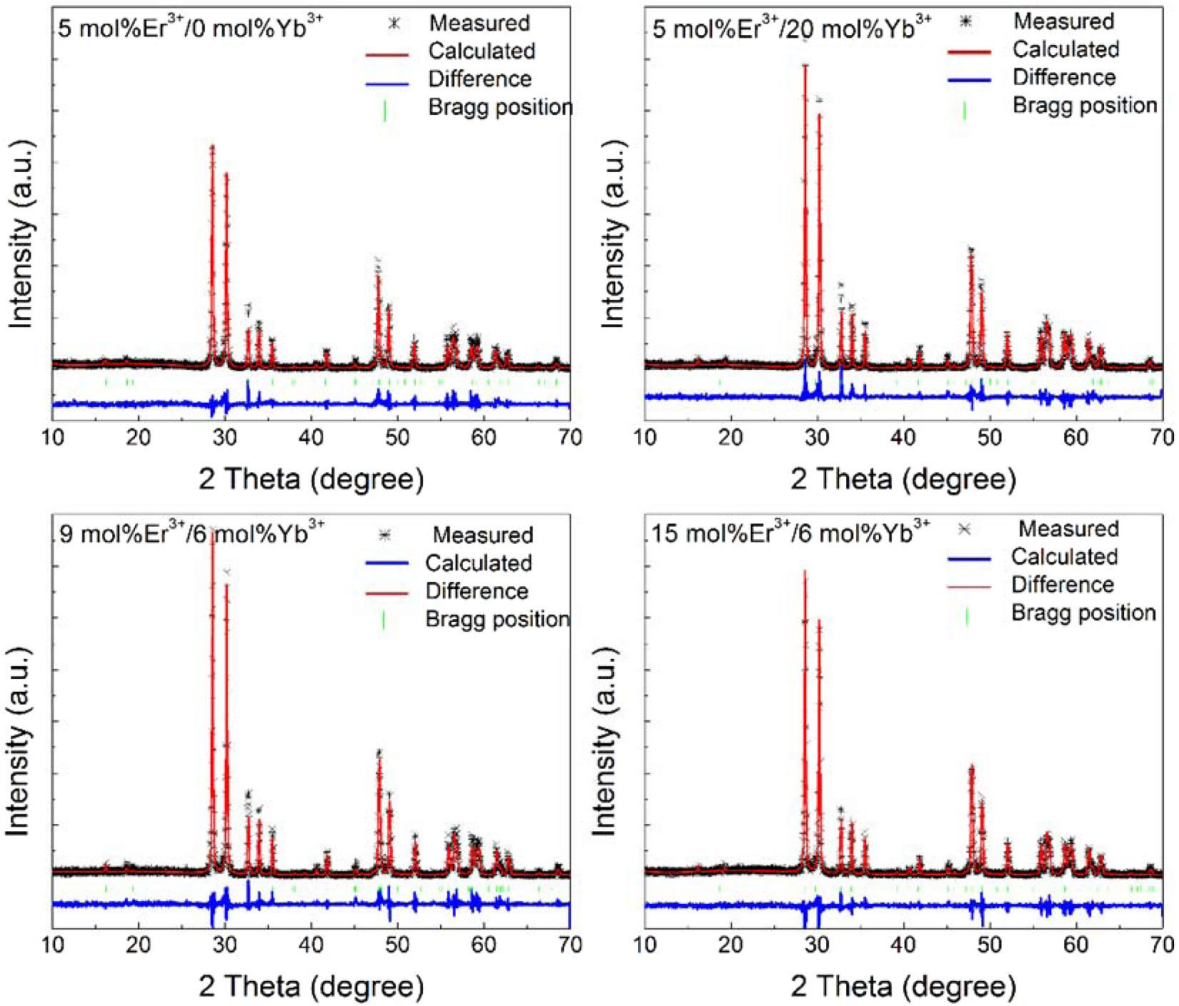
XRD patterns of YNbO_4_: *x* mol% Er^3+^/*y* mol% Yb^3+^ (*x* = 5, *y* = 0 and 20; *y* = 6, *x* = 9 and 15) phosphors. Asterisks represent the experimental data, the red curves accompanying the asterisks show the Rietveld refinement results, the green sticks mark the Bragg reflection positions, and the blue solid lines represent the differences between experimental and calculated data.

In order to identify the crystal structure of the as-prepared phosphors, XRD measurements were performed on all the samples. The results showed that all the samples almost had the same diffraction patterns. To better study the effect of Er^3+^, Yb^3+^ doping concentrations on the crystal structure of YNbO_4_, a Rietveld refinement procedure was carried out with non-commercial software, named General Structure Analysis System (GSAS)^26,27^. Figure 1 shows the XRD Rietveld refinement results of YNbO_4_: *x* mol% Er^3+^/*y* mol% Yb^3+^ (*x* = 5, *y* = 0 and 20; *y* = 6, *x* = 9 and 15) phosphors as representatives. In the refinement processes, the crystallographic data of monoclinic YNbO_4_ (space group of C2/c (15), see JPCDS card No. 83–1319) were used as the initial crystal structure model. The measured, calculated results, Bragg position, and the difference between experimental and calculated diffraction patterns are also shown in Fig. 1. It can be seen that all the diffraction peaks in the experimental data can be well fitted by the Rietveld theoretical model, and the deviation between the experimental and the calculated data are acceptable. The cell parameters and the Rietveld refinement results are shown in Table 1. It can be found that the lattice constants decrease with the increase of both Er^3+^ and Yb^3+^ concentrations, which confirms the disordered lattice induced by the substitution of larger Y^3+^ (0.90 Å) by smaller Er^3+^ (0.88 Å) and Yb^3+^ (0.86 Å). However, all the observed diffraction peaks for those samples satisfy the reflection conditions, and our as-prepared samples are of monoclinic phase.

**Table 1:** Rietveld refinement results and crystal data for the YNbO_4_: *x* mol% Er^3+^/*y* mol% Yb^3+^ (*x* = 5, *y* = 0 and 20; *y* = 6, *x* = 9 and 15).

Formula	Y_0.95_Er_0.05_NbO_4_	Y_0.75_Er_0.05_Yb_0.2_NbO_4_	Y_0.85_Er_0.09_Yb_0.06_NbO_4_	Y_0.79_Er_0.15_Yb_0.06_NbO_4_
Radiation type	Cu Kα_1_ radiation with λ = 1.5406 Å			
*2θ* range	10–70°			
Symmetry	Monoclinic system			
Space group	C2/c (15)			
Cell parameters	a = 7.6116 Å	a = 7.6101 Å	a = 7.6089 Å	a = 7.6068 Å
	b = 10.9423 Å	b = 10.9347 Å	b = 10.9345 Å	b = 10.9323 Å
	c = 5.2938 Å	c = 5.2896 Å	c = 5.2901 Å	c = 5.2890 Å
	α = γ = 90°	α = γ = 90°	α = γ = 90°	α = γ = 90°
	β = 138.411°	β = 138.388°	β = 138.408°	β = 138.405°
	V = 292.666 Å^3^	V = 292.305 Å^3^	V = 292.170 Å^3^	V = 291.988 Å^3^
Reliability factors	χ^2^ = 1.626	χ^2^ = 1.612	χ^2^ = 1.821	χ^2^ = 1.566
	R_wp_ = 23.88%	R_wp_ = 21.99%	R_wp_ = 22.88%	R_wp_ = 21.51%
	R_p_ = 17.40%	R_p_ = 15.65%	R_p_ = 15.77%	R_p_ = 15.58%

### Up-conversion luminescence properties

**Figure 2:**
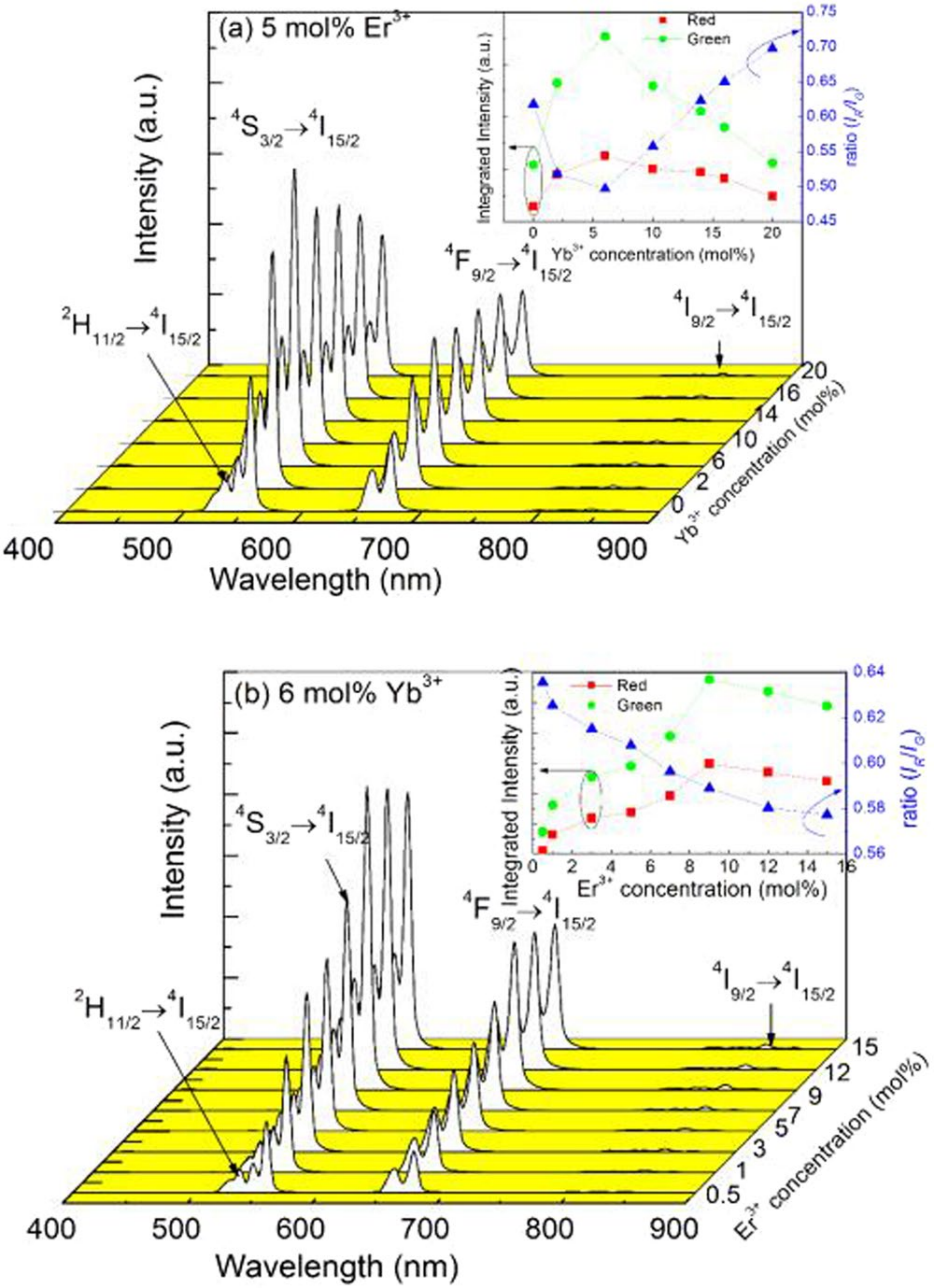
UC luminescence spectra for YNbO_4_ samples doped with various Yb^3+^ concentrations (**a**) and Er^3+^ concentrations (**b**) excited at 1550 nm. The insets show the dependences of the green and the red UC luminescence intensities and the intensity ratio (*I*_*R*_/*I*_*G*_) of red to green UC emissions on Yb^3+^ and Er^3+^ concentrations.

It is well known that the UC luminescence intensity greatly depends on the doping concentrations of RE ions, thus we have studied the effect of Er^3+^ and Yb^3+^ concentrations on the UC luminescence properties excited at 1550 nm wavelength. Er^3+^ and Yb^3+^ concentrations dependent UC luminescence spectra measured at room temperature are displayed in Fig. 2. Figure 2(a) shows the UC luminescence spectra of the as-prepared YNbO_4_ phosphors with fixed Er^3+^ concentration of 5 mol% and various Yb^3+^ concentrations of 0, 2, 6, 10, 14, 16 and 20 mol%. Several characteristic emissions of Er^3+^ can be found in the UC luminescence spectra. Among them, two strong green emissions centered at around 545 and 565 nm can be assigned to the ^2^H_11/2_, ^4^S_3/2_ → ^4^I_15/2_ transitions, and the red emission ranging from 650 to 740 nm originates from ^4^F_9/2_ → ^4^I_15/2_ transition^28,29^. In addition, an extremely weak near infrared emission located at 820 nm corresponding to ^4^I_9/2_ → ^4^I_15/2_ transition can also be observed^30^. The inset of Fig. 2(a) shows the dependences of the green and the red UC emission intensities on Yb^3+^ concentration. It can be seen that the concentration of Yb^3+^ has significant influence on both green and red UC emission intensities. With an increase in Yb^3+^ concentration, both green and red UC emission intensities gradually increase and reach their own maximum values when the concentration of Yb^3+^ is 6 mol%, and then the intensities decline as Yb^3+^ content further increases. Moreover, with increasing Yb^3+^ concentration the integrated intensity ratio (*I*_*R*_*/I*_*G*_) of the red to the green UC emissions exhibits an interesting variation. As can be seen from the inset of Fig. 2(a), the intensity ratio *I*_*R*_*/I*_*G*_ first decreases with an increase in Yb^3+^ concentration, and then significantly increases when the concentration of Yb^3+^ is higher than 6 mol%. The effect of Yb^3+^ concentration on the intensity ratio *I*_*R*_*/I*_*G*_ will be discussed later.

To examine the effect of the concentration of Er^3+^ on the UC luminescence of YNbO_4_: Er^3+^/Yb^3+^ under 1550 nm excitation, the UC luminescence spectra of those samples doped with 6 mol% Yb^3+^ and *x* mol% Er^3+^ (*x* = 0.5, 1, 3, 5, 7, 9, 12 and 15) were measured under an identical experimental conditions and are shown in Fig. 2(b). The intrinsic emissions of Er^3+^ as appearing in Fig. 2(a) are also observed. And when the concentration of Er^3+^ reaches 9 mol%, both green and red UC emission intensities are the strongest, and then decrease with further increase in Er^3+^ concentration, which can be seen in the inset of Fig. 2(b). In addition, from the inset of Fig. 2(b), it can also be found that the intensity ratio *I*_*R*_*/I*_*G*_ decreases with an increase in Er^3+^ concentration in the whole of our studied concentration range, which will be discussed later, too.

**Figure 3:**
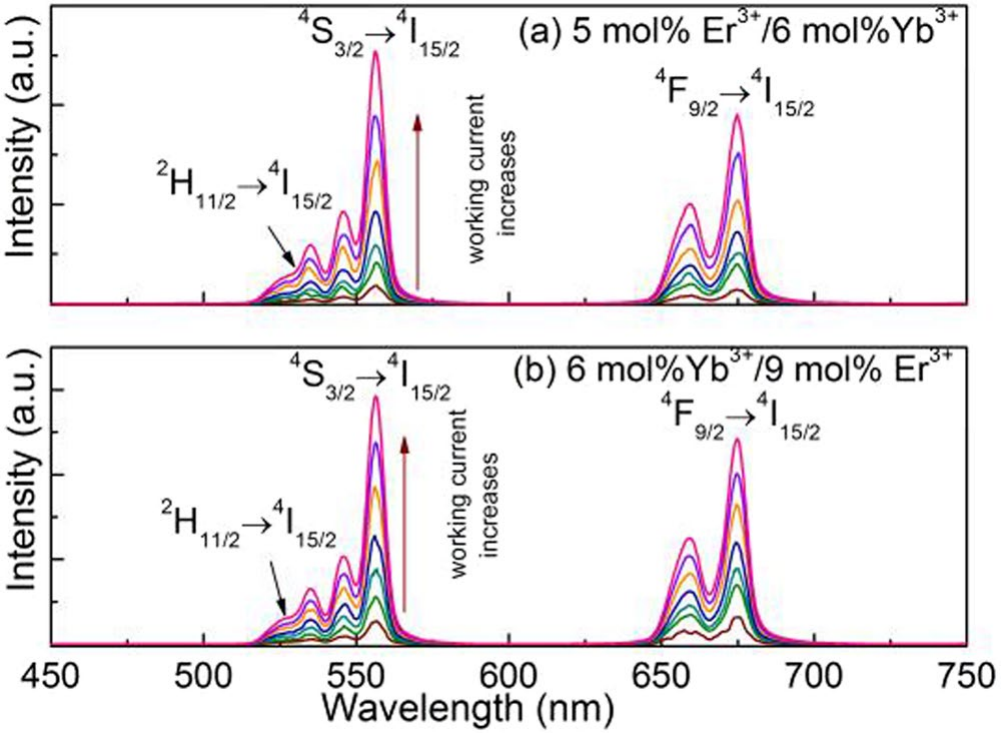
UC luminescence spectra of YNbO_4_: 5 mol% Er^3+^/6 mol% Yb^3+^ (**a**) and YNbO_4_: 9 mol% Er^3+^/6 mol% Yb^3+^ (**b**) phosphors upon 1550 nm excitation at different laser working current.

**Figure 4:**
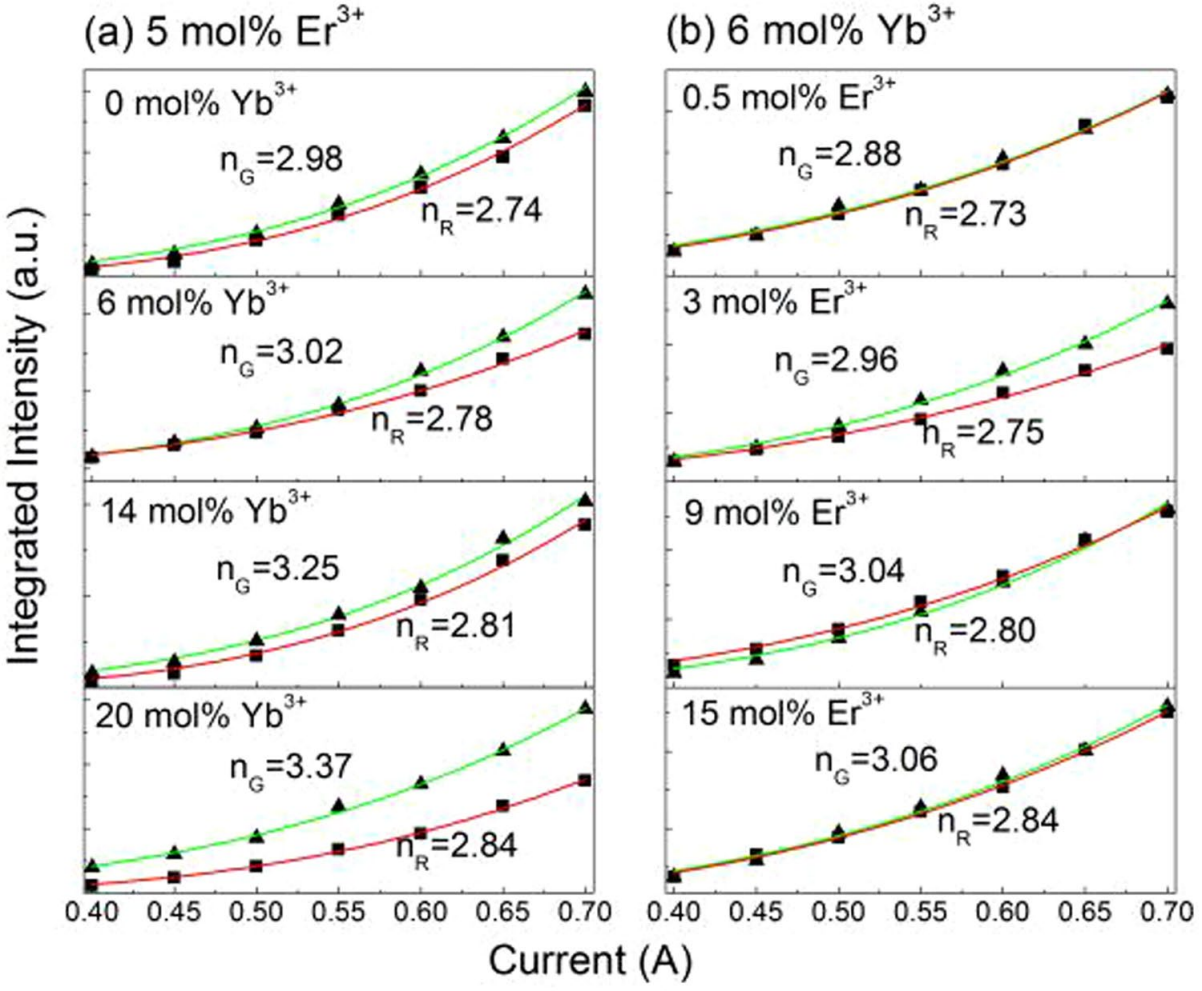
Relationship between the integrated intensities of the green and the red UC emissions and laser working current for different samples, (**a**) 5 mol% Er^3+^/*y* mol% Yb^3+^ (*y* = 0, 6, 14 and 20); (**b**) *x* mol% Er^3+^/6 mol% Yb^3+^ (*x* = 0.5, 3, 9 and 15).

In order to further analyze the UC luminescence processes of Er^3+^ in YNbO_4_: Er^3+^/Yb^3+^ phosphors under 1550 nm excitation, the laser working current dependence of UC luminescence of YNbO_4_: 5 mol% Er^3+^/*y* mol% Yb^3+^ (*y* = 0, 6, 14 and 20) and YNbO_4_: *x* mol% Er^3+^/6 mol% Yb^3+^ (*x* = 0.5, 3, 9 and 15) phosphors were studied. As examples, the UC luminescence spectra for both YNbO_4_: 5 mol% Er^3+^/6 mol% Yb^3+^ and YNbO_4_: 6 mol% Yb^3+^/9 mol% Er^3+^ samples under 1550 nm excitation at different laser working current are shown in Fig. 3(a,b). It should be pointed out that these two samples display the strongest UC luminescence in each set of phosphors in this study. From Fig. 3, it can be seen that the UC luminescence intensities of both samples monotonically increase with an increase of the laser working current. Strong green and red UC emissions were both observed. To further clarify the UC luminescence mechanism of Er^3+^, the integrated intensities of the green and the red UC emissions are calculated separately, and the results for various concentrations of Yb^3+^ and Er^3+^ doped YNbO_4_ phosphors are shown in Fig. 4(a,b). For any unsaturated UC luminescence process, the UC emission intensity (*I*_*up*_) is depended on the laser working current (*i*_LD_), and fulfills the following relation^31^:

1 $${I}_{up}\propto {(a{i}_{{\rm{LD}}}-b)}^{n}$$

where *n* is the number of required photon, *a* and *b* are constants. Equation (1) is used to fit the experimental data depicted in Fig. 4(a,b), and the fitting results are also shown in Fig. 4(a,b). As can be seen, all these *n* values of the red UC emission (*n*_R_) are around 3, indicating that the red UC emissions are dominated by three-photon process for different concentrations of Yb^3+^ and Er^3+^ doped samples. The little differences for those *n*_R_ values may be caused by the calculation error of the integrated intensity and the error in the fitting process. As can be seen in Fig. 4(a), for the green UC emissions the fitted *n* values (*n*_G_) are also around 3. But the value of *n*_G_ continues to grow as Yb^3+^-concentration increases, and it is greater than 3 when Yb^3+^-concentration reaches 14 mol%, indicating much more 1550 nm photons are needed to achieve the green UC emissions in the case of high Yb^3+^-concentration.

**Figure 5:**
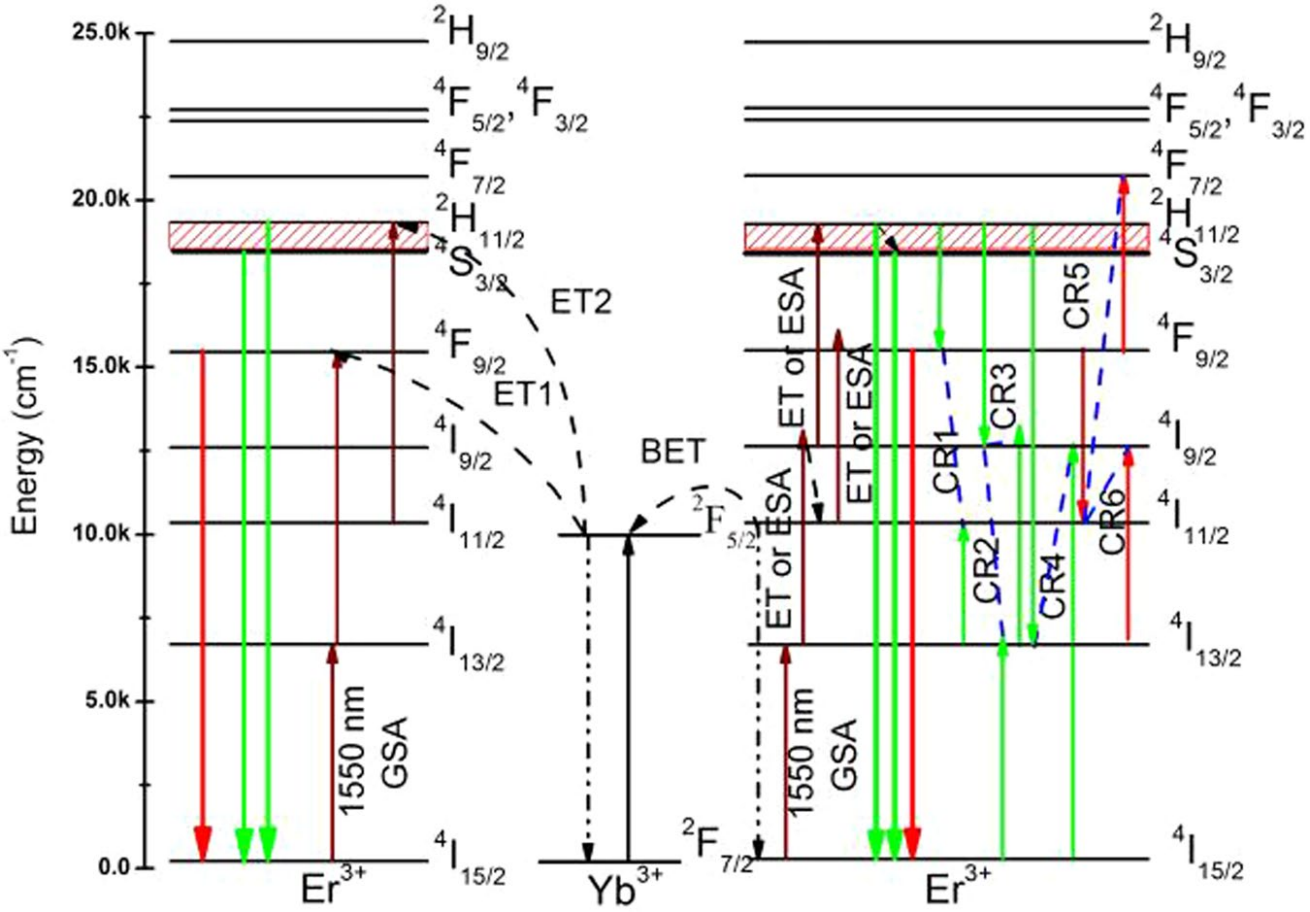
UC luminescence mechanism models of Er^3+^ and possible ET and CR channels of YNbO_4_: Er^3+^/Yb^3+^ phosphors under 1550 nm excitation.

To better describe the concrete UC luminescence processes of Er^3+^ in YNbO_4_: Er^3+^/Yb^3+^ phosphors excited at 1550 nm wavelength, Fig. 5 depicts the simplified energy level diagrams of Er^3+^, Yb^3+^ and the possible energy transfer processes. In principle, there are three possible basic population mechanisms involved in the UC luminescence processes, including energy transfer up-conversion (ETU), ground state absorption (GSA) and excited state absorption (ESA)^10^. As can be seen in Fig. 5, the UC luminescence of Er^3+^ under 1550 nm excitation can be obtained by both direct light absorption of Er^3+^ and ET from Yb^3+^. In this case, for Er^3+^ single-doped sample the population of ^2^H_11/2_ and ^4^S_3/2_ levels can be achieved by successive absorbing three 1550 nm photons via GSA and two ESA or ET (from Er^3+^ to Er^3+^) processes, and then yielding the green UC emissions^24,32^. The possible UC luminescence process of the red UC emission is that Er^3+^ in ^4^I_9/2_ level non-radiatively relaxes to ^4^I_11/2_ level and absorbs one 1550 nm photon or receives an energy transferred from a neighboring Er^3+^, yielding the population of ^4^F_9/2_ level, and then red UC emissions can be achieved.

**Figure 6:**
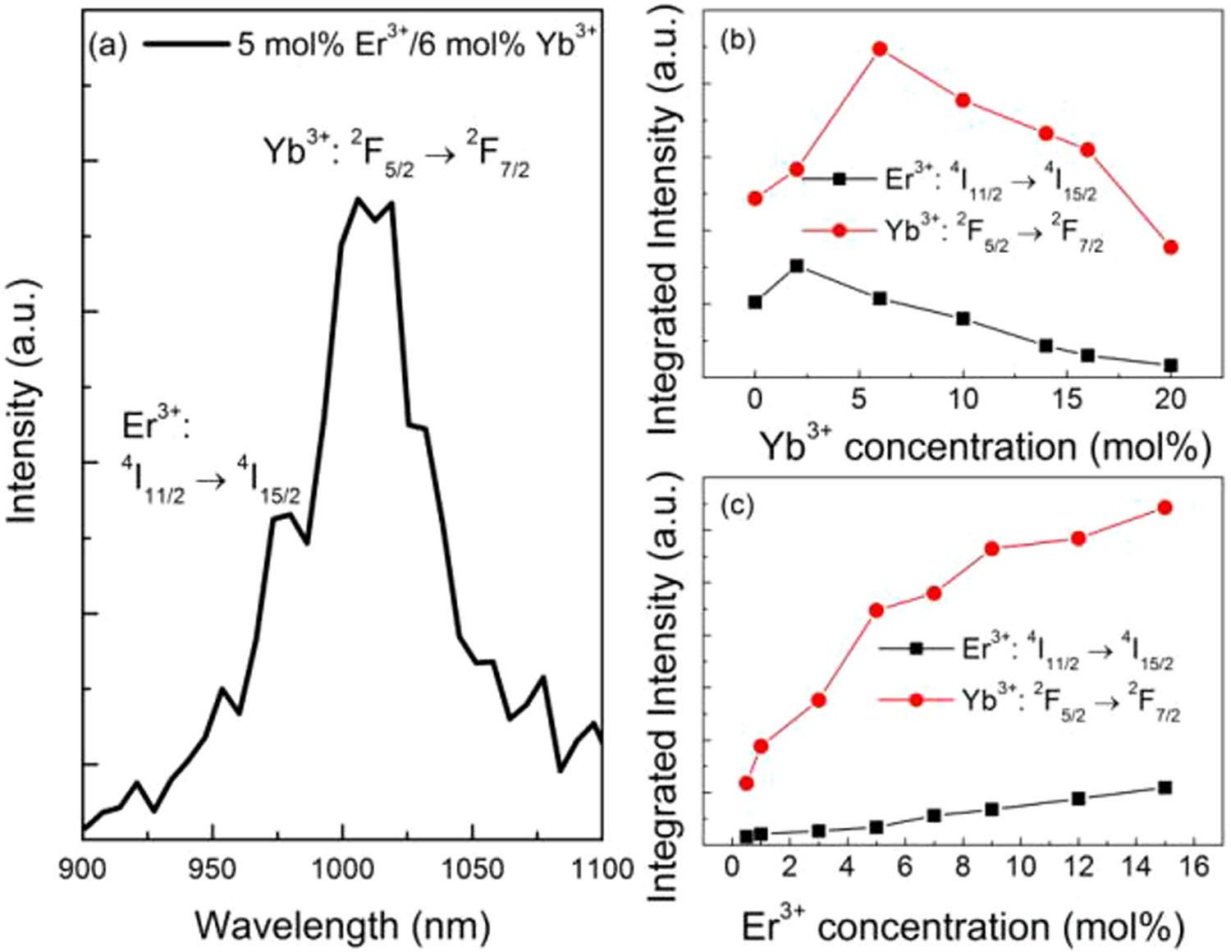
(**a**) Infrared UC luminescence spectra for YNbO_4_: 6 mol% Yb^3+^/5 mol% Er^3+^, and the integrated infrared UC intensities of the samples doped with varied (**b**) Yb^3+^ and (**c**) Er^3+^ concentrations excited at 1550 nm wavelength.

Compared with Er^3+^ mono-doped YNbO_4_ phosphors, introducing Yb^3+^ as a sensitizer can influence the UC luminescence process of Er^3+^ though Yb^3+^ cannot directly absorb the energy of 1550 nm photons. From the inset of Fig. 2(a), it can be found that Yb^3+^ concentration has significant effect on the UC luminescence intensity of Er^3+^. With an increase of Yb^3+^ concentration, the UC luminescence intensity of Er^3+^ first increases and then decreases, which may be caused by the back energy transfer (BET, ^4^I_11/2_ (Er^3+^) + ^2^F_7/2_ (Yb^3+^) → ^4^I_15/2_ (Er^3+^) + ^2^F_5/2_ (Yb^3+^)) process from Er^3+^ to Yb^3+ ^^15^. Er^3+^ ions first absorb the energy of two 1550 photons to achieve the population of ^4^I_9/2_ level, and then complete the population of ^4^I_11/2_ level by non-radiative relaxation process. Because the energy distance between ^2^F_5/2_ level and ^2^F_7/2_ level of Yb^3+^ matches well with that of ^4^I_11/2_ level and ^4^I_15/2_ level of Er^3+^, energy transfer from Er^3+^ to Yb^3+^ can occur. The existence of the BET process can be confirmed by the UC luminescence spectra shown in Fig. 6(b), which were measured under 1550 nm excitation in the near infrared region. As can be seen, the UC emission intensity from Yb^3+^ increases first and then decreases with an increase in Yb^3+^ concentration. Considering the efficient ET process from Yb^3+^ to Er^3+^, Er^3+^ → Yb^3+^ → Er^3+^ ETU process should be an important mechanism for the UC luminescence of Er^3+^ when excited at 1550 nm^11,33,34^. Therefore, the UC luminescence mechanism of Er^3+^ can be described by the following processes. For Er^3+^ and Yb^3+^ co-doped case, Er^3+^ first absorbs two 1550 nm photons populating the ^4^I_9/2_ level and achieves the population of ^4^I_11/2_ level via a following non-radiative relaxation process. Next, Er^3+^ transfers the energy to an adjacent Yb^3+^ via the BET process, and then Yb^3+^ is excited to its excited state ^2^F_5/2_ level. Then, the excited Yb^3+^ transmits part energy to a neighboring Er^3+^, achieving the populations of ^2^H_11/2_/^4^S_3/2_ and ^4^F_9/2_ levels of Er^3+^ via ET2 and ET1 processes, respectively. Finally, the green and the red UC emissions can be achieved. For the green UC emissions, the above-mentioned BET process needs to absorb two 1550 nm photons, and another two 1550 nm photons are needed to be absorbed to accomplish the population of ^4^I_11/2_ level via ET2 process. Thus, four 1550 nm photons are needed for the green UC emission when Yb^3+^ acts as the sensitizer of Er^3+^. Because those values *n*_G_ derived from the fitting process are larger than 3 especially in the case of high concentration of Yb^3+^ doping, it can be concluded that three-photon and four-photon absorption processes co-existent in the green UC luminescence process. That is to say, ETU processes between Er^3+^ and Yb^3+^ and between Er^3+^ ions are co-existent in the green UC luminescence of Er^3+^ under 1550 nm excitation.

Furthermore, the influences of Er^3+^ and Yb^3+^ concentrations on the UC luminescence intensity and the intensity ratio *I*_*R*_*/I*_*G*_ can also be better explained by the energy level diagrams of Er^3+^ and Yb^3+^. For the case of varying Yb^3+^ concentration, when the concentration of Yb^3+^ is not higher than 6 mol%, much more energy can be transferred from Er^3+^ to Yb^3+^ with an increase of Yb^3+^ concentration, which means BET process occurs. Then, higher energy is re-transferred from Yb^3+^ to Er^3+^ via ET1 and ET2 processes, achieving the populations of the green and the red excited state levels ^2^H_11/2_/^4^S_3/2_ and ^4^F_9/2_. However, the increase of the population of ^4^I_13/2_ level will be suppressed compared with that of ^4^I_11/2_ level due to the increased BET process. Finally, the UC luminescence intensity enhances, but the intensity ratio *I*_*R*_*/I*_*G*_ of Er^3+^ decreases. In the case of higher concentration of Yb^3+^ (greater than 6 mol%) doping, the interionic distance between Er^3+^ and Yb^3+^ decreases and those aggregated Yb^3+^ may become quenching center, resulting in the quenching of UC luminescence of Er^3+ ^^35^. In addition, at higher doping concentration of Yb^3+^, the population of ^4^I_11/2_ level may be reduced via BET process much easier than that of ^4^I_13/2_ (as can be seen in Fig. 6(b), the UC luminescence intensity of the emission from ^4^I_11/2_ → ^4^I_15/2_ transition decreases when the concentration of Yb^3+^ reaches 6 mol%), which further affects the populations of ^2^H_11/2_/^4^S_3/2_ and ^4^F_9/2_ levels. Thus, the intensity of the green and the red UC emissions decrease and the ratio *I*_*R*_*/I*_*G*_ increases with an increase of Yb^3+^ concentration.

For variable concentrations of Er^3+^-doped case, the UC luminescence intensity also increases first and then decreases with an increase in Er^3+^ concentration. At higher concentration of Er^3+^, the reduction of UC luminescence intensity of Er^3+^ can be ascribed to the concentration quenching, which may be caused by the non-radiative ET and the cross-relaxation (CR) between Er^3+^ due to the intensified interaction between Er^3+^ for the closer interionic distance. The possible CR processes are shown in Fig. 5^25^. As can be seen, CR1-CR4 processes are responsible for the depopulation of the green emitting levels ^2^H_11/2_ and ^4^S_3/2_, but CR5 and CR6 processes take the responsibility for the depopulation of the red emission level ^4^F_9/2_, which will cause the quenching of the corresponding green and red UC emissions, respectively. With an increase in Er^3+^ concentration, the BET process from Er^3+^ to Yb^3+^ can also be enhanced, and those aggregated Yb^3+^ may be one type of quenching center, which may further induce the quenching of the UC luminescence of Er^3+ ^^34^. In addition, as mentioned above, the intensity ratio *I*_*R*_*/I*_*G*_ decreases with an increase of Er^3+^ concentration in the whole of the studied concentration range, which indicates that the depopulation of the red emitting level ^4^F_9/2_ is much faster than that of the green emitting levels ^2^H_11/2_ and ^4^S_3/2_. As can be seen from Fig. 5, CR1 process could increase the population of ^4^F_9/2_ level, so this CR process can be ignored in the present case. The other CR processes may simultaneously influence the population of the green and the red emitting levels assisted by other ET, energy absorption or non-radiative transition processes.

### Temperature sensing properties

Temperature is an important physical quantity in both science and industrial fields, and it has a great influence on the spectroscopic properties of the luminescent materials. However, it is difficult to monitor the actual temperature of the materials via traditional contact measurement due to the interference from the introduced thermometers. Thus, it is necessary to find a non-contact method to measure the internal temperature of one material.

**Figure 7:**
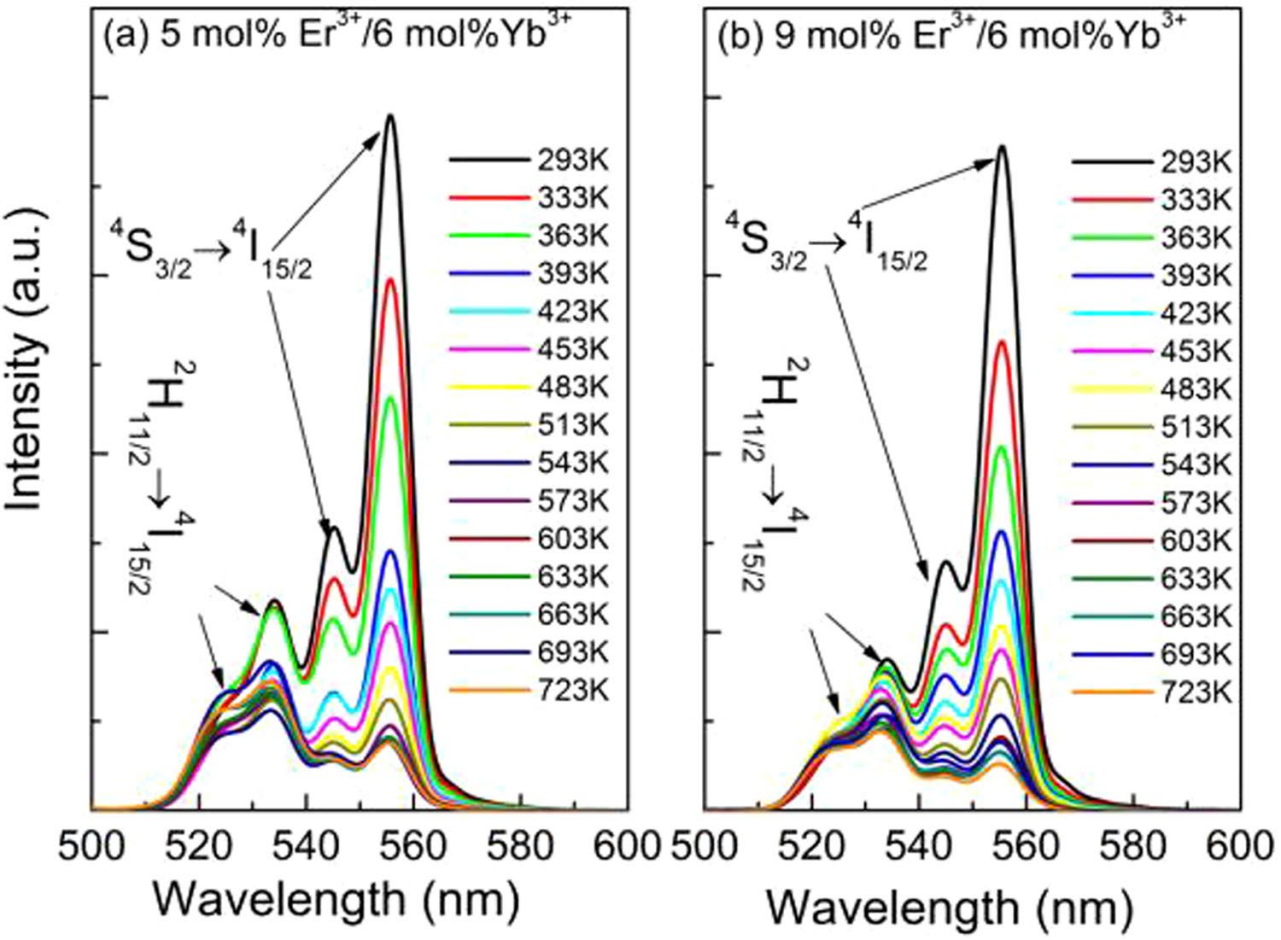
Temperature dependent UC luminescence spectra for (**a**) YNbO_4_: 5 mol%Er^3+^/6 mol% Yb^3+^ and (**b**) 6 mol% Yb^3+^/9 mol% Er^3+^ under 1550 nm excitation.

**Figure 8:**
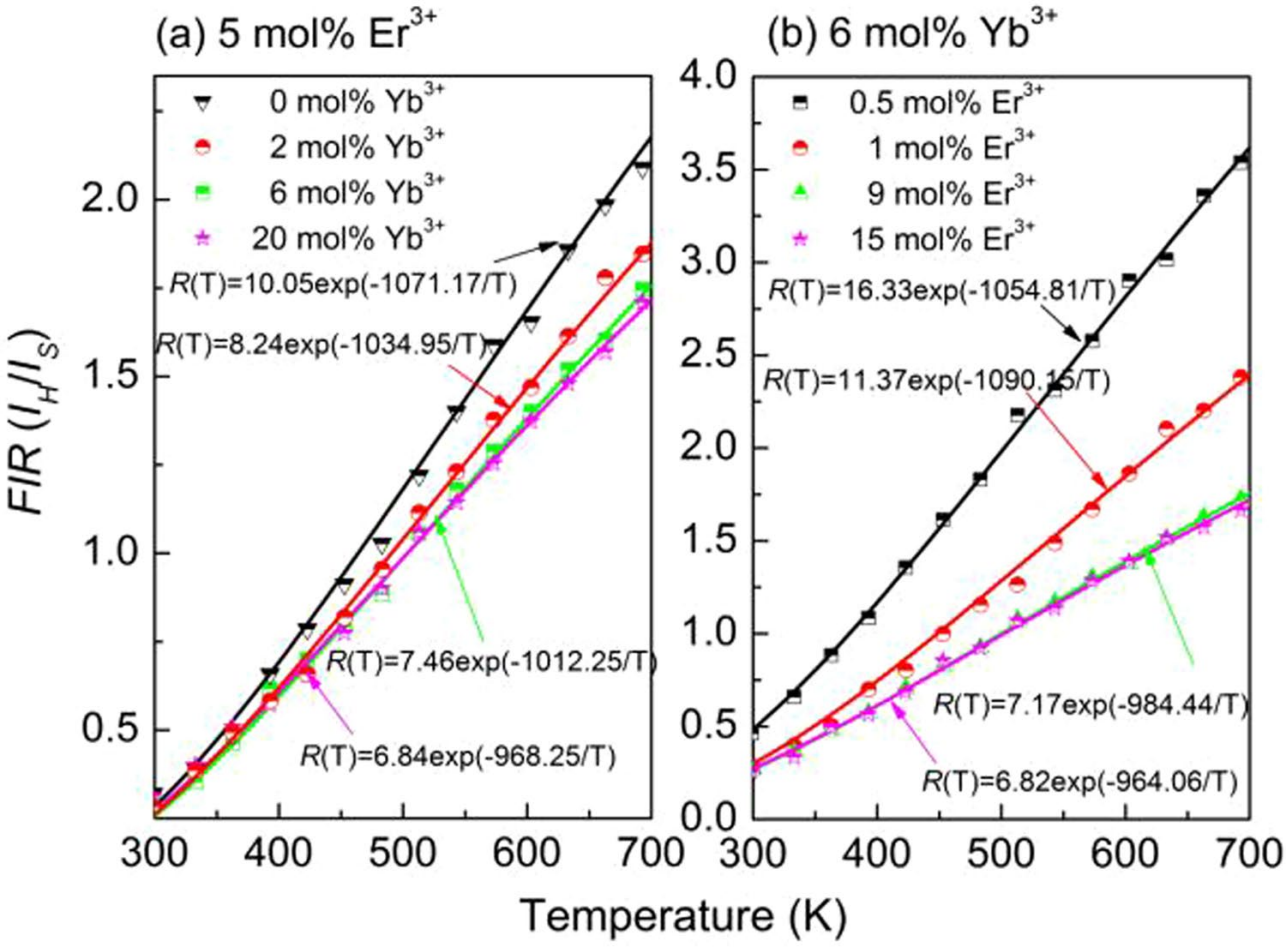
The relationships between the intensity ratios *R* and the sample temperature for YNbO_4_ phosphors doped with 5 mol% Er^3+^/*y* mol% Yb^3+^ (*y* = 0, 2, 6 and 20) (**a**) and *x* mol% Er^3+^/6 mol% Yb^3+^ (*x* = 0.5, 1, 9 and 15) (**b**).

The energy distance between the two levels ^2^H_11/2_ and ^4^S_3/2_ of Er^3+^ is small and these two levels exist in a state of thermal equilibrium, thus their populations fulfill the Boltzmann’s distribution^36^. And then the *FIR* of these two green emissions is temperature dependent. Therefore, Er^3+^ can be used as temperature sensing unit to achieve temperature detection via probing the *FIR* of the two green emissions. According to the Boltzmann’s distribution theory, the dependence of the *FIR* value (*R, I*_*H*_*/I*_*S*_) on temperature for Er^3+^-doped samples can be expressed as follows^37^:

2 $$R(T)=\frac{{I}_{H}}{{I}_{S}}=A\,\exp (\frac{-{\rm{\Delta }}E}{kT})$$

where *I*_*H*_ and *I*_*S*_ are the integrated fluorescence intensities of the emissions from ^2^H_11/2_ → ^4^I_15/2_ and ^4^S_3/2_ → ^4^I_15/2_ transitions, respectively. *ΔE* is the energy distance between ^2^H_11/2_ and ^4^S_3/2_ levels, *k* is Boltzmann’s constant, and *T* is absolute temperature. To investigate the optical temperature sensing behavior of Er^3+^ in YNbO_4_: Er^3+^/Yb^3+^ phosphors, the UC luminescence spectra of the samples doped with 5 mol% Er^3+^/*y* mol% Yb^3+^ (*y* = 0, 2, 6 and 20) and *x* mol% Er^3+^/6 mol% Yb^3+^ (*x* = 0.5, 1, 9 and 15) were measured at various temperatures ranging from 293 K to 723 K. Figure 7 shows the temperature dependent UC luminescence spectra for the samples with the maximum UC luminescence intensity of the two groups in our study. As can be seen, the two green emission bands originated from ^2^H_11/2_, ^4^S_3/2_ → ^4^I_15/2_ transitions can be observed, and the overall luminescence intensity decreases with an increase of temperature, indicating temperature dependent luminescence quenching occurs. Generally, non-radiative relaxation, energy transfer and crossover process are the main three reasons which may cause the temperature quenching of luminescence^16,25^. With an increase of sample temperature, the non-radiative relaxation rate of the emitting levels, the energy transfer probability originating from the emitting levels, and the crossover process between the emitting levels of the luminescent centers and the charge transfer band of them or the absorption band of the matrix in high energy region will be enhanced, which will result in the quenching of the luminescence intensity. Similar to our previous research results, the non-radiative relaxation process would be the main mechanism for the temperature dependent UC luminescence quenching of Er^3+^ in the present case^16,25^. In addition, it can also be found that the peak positions of them almost unchanged but the relative intensity and the changing trend of each green UC emission are different with an increase of temperature. It can be seen that the intensity of the green UC emission originated from ^2^H_11/2_ → ^4^I_15/2_ transition first decreases slightly and then remains approximately unvaried. However, the intensity of the emission from ^4^S_3/2_ → ^4^I_15/2_ transition monotonically decreases with an increase of temperature. However, the intensity ratios *R* monotonically increases with increasing the sample temperature, which can be seen from Fig. 8(a,b). As can be seen in Fig. 8(a,b), all the experimental data can be fitted well by equation (2). The confirmed equations for different samples are also shown in Fig. 8(a,b). As can be seen, all of the values of *ΔE/k* are around 1000. The corresponding *ΔE* value was 694.5 cm^−1^, which is in good agreement with the value (713.8 cm^−1^) derived from the measured UC luminescence spectra in Fig. 2. This fact means that equation (2) can explain well the temperature dependence of the *FIR* of the two green UC emissions.

With regard to the application in optical temperature sensing, it is extremely important to be aware of the temperature sensing sensitivity. Usually, the sensor sensitivity can be defined as the change rate of *R* per unit temperature and can be expressed as follows^38^:

3 $$S(T)=\frac{dR(T)}{dT}=A\,\exp (\frac{-{\rm{\Delta }}E}{kT})(\frac{{\rm{\Delta }}E}{k{T}^{2}})$$

**Figure 9:**
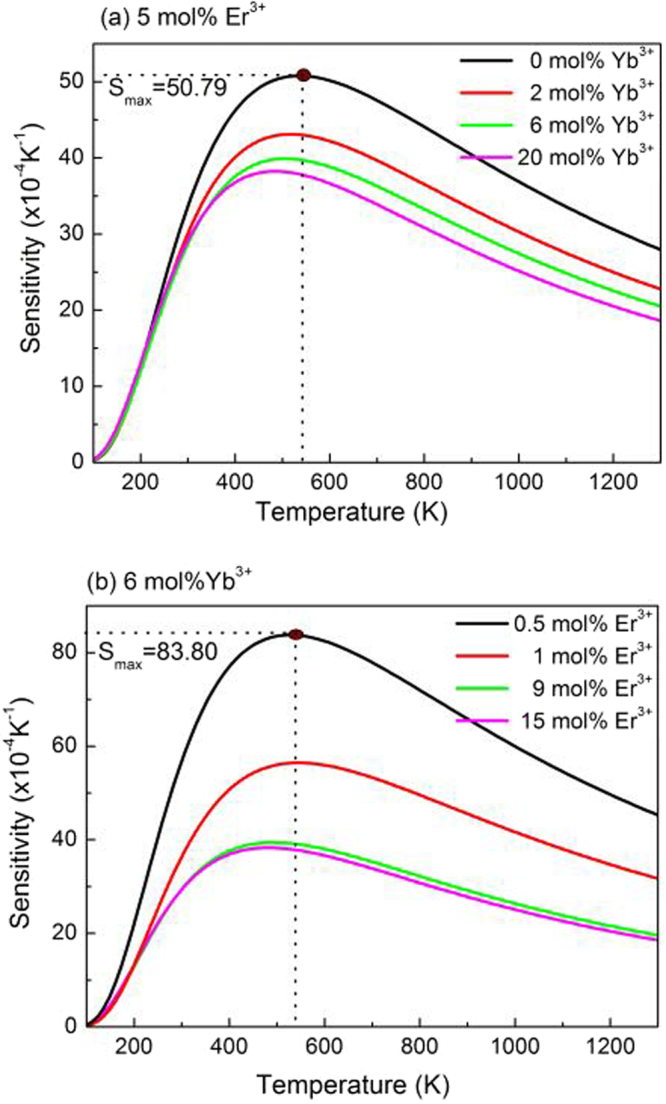
Sensitivity curves for YNbO_4_: Er^3+^/Yb^3+^ phosphors doped with 5 mol% Er^3+^ and 0, 2, 6, 20 mol% Yb^3+^ (**a**); 6 mol% Yb^3+^ and 0.5, 5, 9, 15 mol% Er^3+^ (**b**).

By taking the values of *A* and *ΔE/k* derived from the fitting processes in Fig. 8(a,b) into equation (3), the sensitivities of the samples were calculated and are shown in Fig. 9(a,b). It can be seen that the sensitivities of all the samples have the same changing trend, and all of them are dependent on the sample temperature. With an increase of sample temperature, the sensitivity first increases and reaches the maximum value at around 536 K, and then dramatically decreases. In addition, it can also be found that the concentrations of Er^3+^ and Yb^3+^ have a great effect on the temperature sensing sensitivity. The sensitivity decreases with an increase of the doping concentrations of Er^3+^ and Yb^3+^. Similar phenomenon were observed in YNbO_4_: Er^3+^ and NaGdTiO_4_: Er^3+^, Yb^3+^ phosphors in our previous studies, which was attributed to the different optical transition rate of Er^3+^ in different samples doped with various concentrations of Er^3+ ^^22,39^. Therefore, if we want to obtain high temperature sensitivity, the doping concentrations of Er^3+^ and Yb^3+^ should be further reduced. However, when the concentration is too low, the luminescence of the sample is too weak, which is likely to cause large spectral measurement errors. Therefore, we still need to select the doping concentration of RE ions properly in practical applications. In our present study, the maximum sensitivity of Er^3+^ is 83.80 × 10^−4^ K^−1^ for 0.5 mol% Er^3+^/6 mol% Yb^3+^ doped sample.

### Laser-induced thermal effect

The UC luminescence process is often accompanied by heat generation, especially when the luminescence materials are excited at long wavelength laser source with high power density. The laser irradiation can result in an elevation of the sample temperature, and further bring temperature dependent effect on the spectroscopic properties of the luminescence materials, thus it is necessary to study the laser-induced thermal effect of the UC luminescence materials.

**Figure 10:**
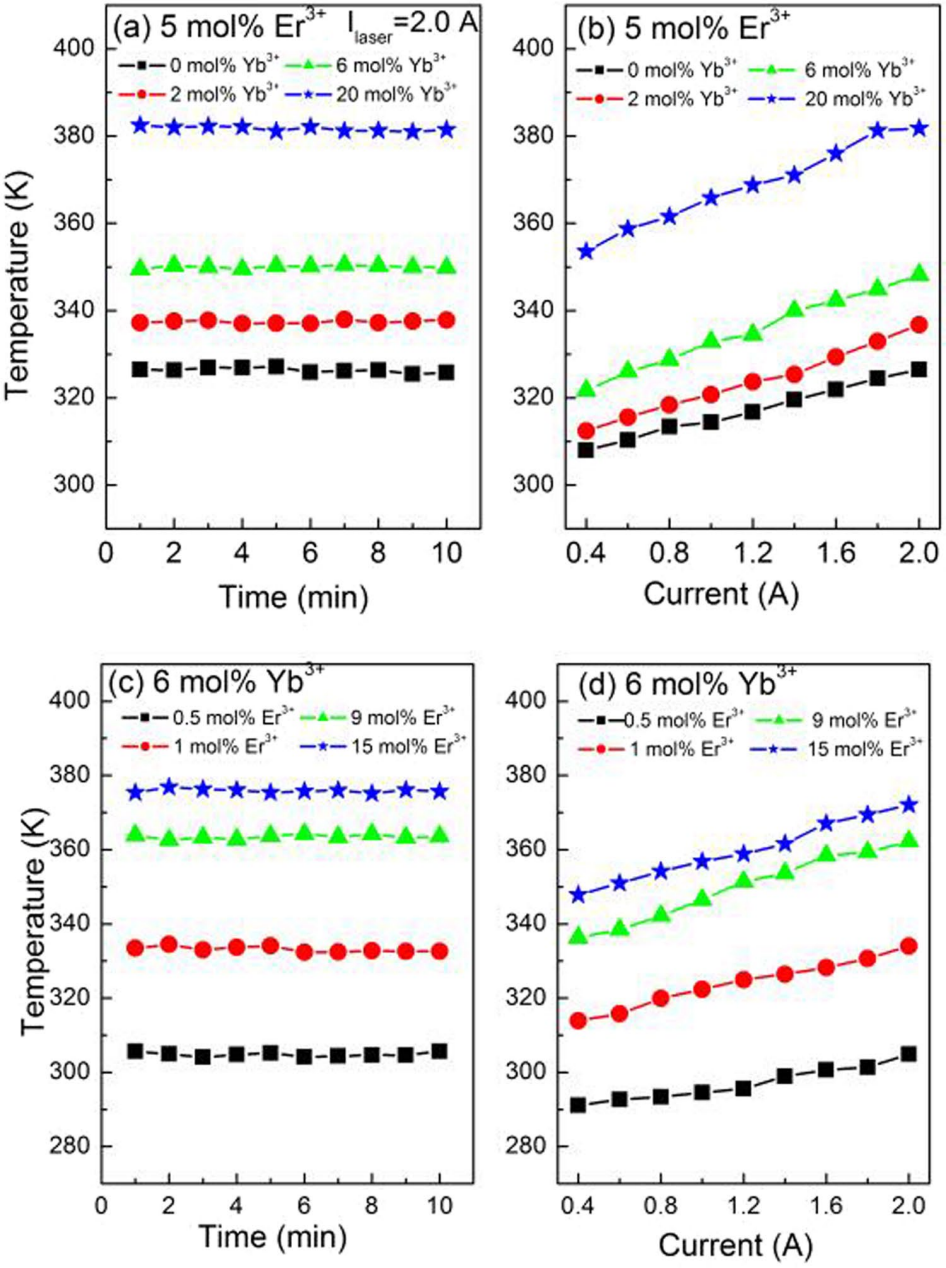
Dependence of sample temperature on the 1550 nm laser irradiation time and working current for the sample with different Er^3+^ and Yb^3+^ concentrations.

To understand the thermal effect induced by 1550 nm laser irradiation on the samples, the UC emission spectra of YNbO_4_: Er^3+^/Yb^3+^ phosphors with various doping concentrations of Er^3+^ and Yb^3+^ were measured when these samples were continuously irradiated by 1550 nm laser with working current of 2.00A for 10 minutes. The integrated intensity ratios of the two green emissions were calculated according to the UC luminescence spectra, and the sample temperatures were derived by taking the integrated intensity ratios into equation (1). Figure 10(a,c) show the dependence of the sample temperature on the irradiation time of 1550 nm laser for the Er^3+^ and Yb^3+^ concentration-varied samples, the working current of 1550 nm laser is fixed at 2.00 A. It can be seen that the temperatures of all the samples change very slightly with irradiation time, indicating that all the samples can quickly reach thermal equilibrium. In addition, it can also be found that the sample temperatures are higher than the room temperature, suggesting the existence of laser-induced thermal effect. Figure 10(b,d) show the dependence of the sample temperatures on the working current of 1550 nm laser for the Er^3+^ and Yb^3+^ concentration-varied samples. It can be seen that the sample temperature increases with an increase of the laser working current, indicating significant laser-induced thermal effect in high power density (corresponding to large working current of 1550 nm laser) pumped case. Moreover, it is also observed that the higher the Er^3+^ (Yb^3+^) concentration, the higher the sample temperature.

## Conclusion

YNbO_4_ phosphors co-doped with various concentrations of Er^3+^ and Yb^3+^ were synthesized via a high temperature solid-state reaction method. XRD results indicated that monoclinic YNbO_4_ phosphors were obtained. The concentration dependent UC luminescence properties were analyzed and it was found that the UC luminescence intensities greatly depended on the doping concentrations of Er^3+^ and Yb^3+^. By the analysis of the laser working current dependent UC luminescence spectra, it was confirmed that three-photon processes were dominated in the red UC emissions, and three-photon and four-photon processes co-existed in the green UC emissions under 1550 nm excitation. In addition, the study of the temperature sensing properties showed that the temperature sensing sensitivity is concentration dependent, and the lower concentrations of Er^3+^ and Yb^3+^, the higher temperature sensitivity. Furthermore, the YNbO_4_: Er^3+^/Yb^3+^ phosphors can quickly reach thermal equilibrium excited by 1550 nm laser with high laser power density, and the sample temperature induced by 1550 nm laser irradiation was significantly dependent on both the RE doping concentration and the laser working current. The above results demonstrate that the concentrations of Er^3+^ and Yb^3+^ have significant influences on the UC luminescence processes, the temperature sensitivity of Er^3+^ and the laser-induced thermal effect of YNbO_4_: Er^3+^/Yb^3+^ phosphors. Our results can provide a good reference for choosing proper doping concentrations of RE ions in the study of Er^3+^ related UC luminescence and temperature sensing.
